# Role of hospitals in recovery from COVID-19: Reflections from hospital managers and frontliners in the Eastern Mediterranean Region on strengthening hospital resilience

**DOI:** 10.3389/fpubh.2022.1073809

**Published:** 2023-01-18

**Authors:** Hamid Ravaghi, Merette Khalil, Jehan Al-Badri, Antoinette Vanessa Naidoo, Ali Ardalan, Hamidreza Khankeh

**Affiliations:** ^1^Department of Universal Health Coverage and Health Systems, WHO Regional Office for the Eastern Mediterranean, Cairo, Egypt; ^2^Health Emergencies Program, WHO Regional Office for the Eastern Mediterranean, Cairo, Egypt; ^3^Division of Emergency Medicine, University of Cape Town, Cape Town, South Africa; ^4^Health in Emergency and Disaster Research Center, University of Social Welfare and Rehabilitation Sciences, Tehran, Iran; ^5^Department of Clinical Science and Education, Karolinska Institute, Stockholm, Sweden

**Keywords:** hospital, resilience, recovery, health emergency and disaster risk management, COVID-19

## Abstract

**Background:**

COVID-19 highlighted the critical role that hospitals play throughout the prolonged response and continuous recovery stages of the pandemic. Yet, there is limited evidence related to hospitals in the recovery stage, particularly capturing the perspectives of hospital managers and frontliners in resource-restrained and humanitarian settings.

**Objective:**

This paper aims to capture the perspectives of hospital managers and frontliners across the Eastern Mediterranean Region on (1) the role of hospitals in recovering from COVID-19, (2) Hospitals' expectations from public health institutions to enable recovery from COVID-19, (3) the Evaluation of hospital resilience before and through COVID-19, and (4) lessons to strengthen hospital resilience throughout the COVID-19 recovery.

**Methods:**

A multi-methods approach, triangulating a scoping review with qualitative findings from 64 semi-structured key-informant interviews and survey responses (*n* = 252), was used to gain a deeper context-specific understanding. Purposeful sampling with maximum diversity supported by snowballing was used and continued until reaching data saturation. Thematic analysis was conducted using MAXQDA and simple descriptive analysis using Microsoft Excel.

**Findings:**

In recovering from COVID-19, hospital managers noted hospitals' role in health education, risk reduction, and services continuity and expected human resource management, financial and material resource mobilization, better leadership and coordination, and technical support through the provision of updated clinical evidence-based information from their public health institutions. Qualitative findings also indicated that hospital managers attributed considerable changes in hospitals' resilience capacities to the pandemic and suggested that strengthening hospitals' resilience required resilient staff, sustainable finance, and adaptive leadership and management.

**Conclusion:**

Hospitals are the backbone of health systems and a main point of contact for communities during emergencies; strengthening their resilience throughout the various stages of recovery is critical. Hospitals cannot be resilient in silos but rather require an integrated-whole-of-society-approach, inclusive of communities and other health systems actors.

## 1. Background

Hospitals are the backbone of health systems and a main point of contact for communities during emergencies; it is, therefore, imperative to ensure their continued functionality, safety, and resilience (1). “Hospital resilience” can be conceptualized by its six interdependent components (1) space, (2) stuff, (3) staff, (4) systems, (5) strategies, and (6) services), four resilience capacities (absorptive, adaptive, transformative, and learning), resulting in the primary outcome where resilient hospitals fulfill their most essential functionality then recover to its original state or a new adaptive state in a timely and efficient manner (2). In many conflict-affected or fragile health systems, where shocks are chronic and prolonged, resilience is day-to-day, with daily opportunities to adapt and transform in response to complex challenges and various simultaneous types of hazards (3). In this light, hospital resilience comprises both everyday resilience strengthened during routine operations as well as event-based emergency preparedness and response which require surge capacity (1). Hospital (and health systems) resilience occurs through each of the disaster risk management (DRM) cycle or stages of prevention, preparedness, response, and recovery (PPRR) (1). In many public health emergencies, the stages of response and “early recovery” are often overlapping with numerous interventions needed to rapidly stabilize and address the immediate needs of the population during a crisis (4). Scholars note the importance of hospitals' functionality (particularly emergency units) during the first 3 days highlighting the “72-hour golden window” to optimize survivorship following emergencies (5, 6). On the other hand, the pandemic has intertwined the response and recovery stages over 3 years as hospitals continued responding to COVID-19 while recovering to resume the provision of their services (7). Furthermore, hospitals are frontlines during public health emergencies, ensuring their immediate recovery and functionality is therefore central to both health systems and community resilience (8, 9). Despite the critical role hospitals play in DRM, across the literature, there is limited evidence related to hospital's resilience particularly in the recovery stage (2).

According to the United Nations Office for Disaster Risk Reduction, recovery is defined as: “The restoring or improving livelihoods and health, as well as economic, physical, social, cultural and environmental assets, systems and activities, of a disaster-affected community or society, aligning with the principles of sustainable development and “build back better,” to avoid or reduce future disaster risk” (4). The recovery stage encompasses early recovery, leading to short-, medium-, and long-term rehabilitation, and finally reconstruction, which eventually closes the PPRR cycle back to prevention and preparedness. Moreover, the “build back better” (BBB) is a core principle of recovery and offers the opportunity to build back more resilient hospitals, health systems, communities, and societies more broadly. A study from the natural-disaster-prone Caribbean region described an efficient approach post-disaster “resilient recovery highlighting three dimensions to the BBB approach: (1) building back *stronger* (reconstructed infrastructure can resist more intense events), (2) building back *faster* (income, assets, consumptions, and services are restored as early as possible), and (3) building back more *inclusively* (reaching the poorest, most exposed, and most vulnerable) (10). Another interpretation of the BBB approach brought together six dimensions of communities (people, place, planet, peace, prosperity, and participation), centering governance and equity, and highlighting the impacts of healthy cities on the health and wellbeing of communities, which ultimately result in urban, sustainable, economic, human and social development (11). Furthermore, in understanding the role of hospitals in recovery and resilience, it is important to consider the multi-sectoral nature of recovery and the interdependence and interlinkages between hospitals, health systems, and community resilience (12). Resilient hospitals contribute to building stronger and more resilient health systems, and healthy communities, and ultimately impact sustainable development (7). Beyond fulfilling their primary function in service delivery, hospitals also play a critical role in essential public health functions (EPHF) such as disaster risk reduction and also contribute to social, economic, and community development, and environmental sustainability (2).

The Eastern Mediterranean Region (EMR) reports the highest number of humanitarian emergencies exacerbating pressures on health systems which often face multiple types of hazards simultaneously. The EMR is a highly diverse Region, with 6 high-income countries (Group 1), 4 upper-middle income, 7 lower-middle income (Group 2), and 5 low-income (Group 3) (Box 1) (13). In the EMR, there are ~740,000 hospital beds, with 80% in the public sector (1). At the beginning of the pandemic, hospitals in the Region were challenged, in learning and responding to a new virus with many countries also facing humanitarian emergencies; as the years progressed, these challenges were constantly evolving (14). In the first months of the COVID-19 response (and early recovery), health workers, hospital managers, and policymakers faced fear and anxiety due to the high rates of infections, limited and conflicting evidence-based guidelines, and misinformation and stigmatization of the virus and hospitals (14). Hospitals suffered from financial losses due to disrupted health services and increased costs, along with shortages of health workers (specifically ICU specialists/staff), and disrupted supply chains exacerbating the global shortages of sufficient PPEs, testing kits, and supplies (1). These shortages and limited testing capacities resulted in delays in diagnosing and confirming suspected cases which contributed to designated hospitals being overwhelmed, inefficient use and wastage of resources, and in some cases preventable infections and deaths among patients and health workers (1). Across the Region, the highest reported challenges were the shortages of staff and Personal Protective Equipment (PPEs) (14). A regional study further highlighted the adaptability of EMR hospitals in addressing complex challenges to maintain operations, respond to emergencies, and protect patients and staff, while also continuously evolving to strengthen their readiness for subsequent surges and plan for recovery (7, 14). Throughout the pandemic, hospitals (and health systems) needed to be resilient, continuously learning, absorbing, adapting, and transforming to ensure the safe and continuous delivery of critical services during emergencies (2, 13, 15). Hospitals exhibited these four resilience capacities throughout the prolonged COVID-19 response and overlapping recovery stages.

**Box 1:**
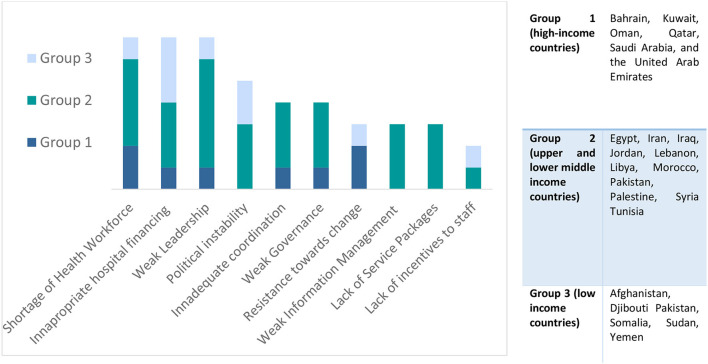
Challenges faced by hospitals in the EMR by country-groups, according to WorldBank 2022 income-classification (from 2019 survey, unpublished by WHO/EMRO).

Across the literature, evidence on hospital resilience remains nascent and generally concentrated in the Global North, with few studies from the EMR and fragile and conflict-affected settings (2). Literature across both hospital and health systems resilience offers divergent and inconsistent definitions and frameworks for conceptualization with limited evidence on its operationalization and evaluation through the stages of PPRR (2). Moreso, evidence on health systems and hospital resilience focuses on the preparedness and response stages, with limited evidence on recovery (2, 16, 17). While the role of hospitals in recovering from emergencies is generally assumed, it remains under-documented (2). Further to this, across the literature on hospital resilience, limited research qualitatively captures the reflections of hospital managers, policymakers, and frontline emergency response managers in resource-restrained and conflict-affected settings. Addressing these research gaps, this paper aims to capture the perspectives of hospital managers (HMs) across the EMR on:

(1) The role of hospitals in recovering from COVID-19,
(2) Hospitals' expectations from public health institutions to enable recovery from COVID-19,
(3) Evaluation of hospital resilience before and through COVID-19,
(4) Lessons to strengthen hospital resilience throughout the COVID-19 recovery.

## 2. Methods

Due to the dearth of literature in the early stages of the pandemic, particularly from the EMR, this multi-methods study triangulated findings from three sources: literature review, online survey, and in-depth semi-structured key informant interviews (KIIs), to comprehensively capture hospitals' diverse and complex experiences in combatting and recovering from COVID-19 from the Region. For the purposes of this paper, we consider the intertwined phase between response and early recovery as recovery.

This paper is a derivative of a large mixed-methods regional research, which occurred over two phases: firstly, at the onset of the COVID-19 pandemic in March 2020 related to hospitals' experiences (challenges, interventions, and lessons learned) in combatting COVID-19, and secondly, 2 years later, related to hospitals' subsequent resilience throughout the prolonged response and recovery phases. During the first phase of the research, qualitative data was gathered from participants regarding five areas: (1) challenges, (2) interventions, (3) lessons learned in combatting COVID-19, (4) the role of hospitals in the recovery, and (5) hospitals' expectations from their public health enabling their response and recovery. During the second stage, participants were asked about (1) their conceptualization, (2) interventions for operationalizing, and (3) strategies for evaluating their hospital's resilience, along with (4) the lessons learned in strengthening hospital resilience throughout the pandemic. This qualitative paper synthesizes the findings from parts 4 and 5 of stage 1 and part 4 of stage 2, using qualitative content analysis, to address the prominent research gap on recovery and resilience, particularly from the EMR. The findings of the other stages, including the literature review, can be found in following references (2, 7, 14).

Regarding the first objective, a broad question was intentionally asked regarding the role of hospitals in recovery, and responses were tiered as they related to spheres of influence (role on the hospital itself, community, health system, society, globally or the planet). Regarding the second objective, for the purposes of this research, public health institutions were divided into national and international. Hospitals were asked about their expectations from (1) the Ministries of Health (MOH) as the leading health systems coordinator at the national level and (2) the World Health Organization (WHO), as the United Nations' leading organization on global health. Regarding the third objective, we qualitatively evaluated hospital managers' perceptions and experiences of their hospitals' resilience before and after COVID-19, using Likert-scale questions in an online survey triangulated with open-ended questions across key informants and survey modalities. Ten statements related to hospitals' responses across the DRM cycle captured the hospital's capacities to absorb, adapt, transform, and learn, in accordance with the definition of hospital resilience presented in the background. Of these ten statements, eight were either directly or indirectly related to the recovery stage including early recovery (which starts during the response stage) and learning (which occurs through the prevention and preparedness stages). Finally, for the fourth objective, we asked hospital managers regarding their top 1–3 tips or lessons to their peers on strengthening hospital resilience. Most of their responses were related to hospital resilience components offering a complementary perspective to the survey which predominantly captured capacities.

### 2.1. Study design and data collection

To complement the limited findings from the literature review and gain a deeper understanding of the context-specific and diverse challenges and experiences faced by hospitals in the EMR, this study utilized a qualitative methodology, based on data from KIIs and open-ended survey questions. For both modalities, responses were collected anonymously and voluntarily during both stages of data collection. KIIs provided their informed verbal and written consent after receiving all relevant information about the project, a detailed consent form, and a copy of the interview topic guide. Survey respondents read an introduction to study objectives and an overview of ethical considerations before accessing the questions; all responses were collected anonymously and voluntarily. This study received ethical approval from the Regional Ethical Review Committee of the World Health Organization's Eastern Mediterranean Regional Office, which permits research to be conducted in the 22 countries of the Region.

Semi-structured in-depth key informant interviews were conducted during the two phases of the research: firstly, between Jul–Oct 2020 and secondly between Nov 2021–Feb 2022. For each stage, a topic guide for semi-structured interviews was created and an online questionnaire using GoogleForms was developed, piloted, and disseminated widely through WHO country offices to key national stakeholders, their staff, and professional networks *via* email and social media platforms such as WhatsApp. All study tools were reviewed by a small team of multi-disciplinary global and regional experts in the fields of health systems, hospital management, emergency response, and disaster management. The study tools were piloted and modified accordingly.

In all stages, purposeful sampling with maximum diversity was used to recruit interviewees ensuring maximum variation. WHO country offices recruited participants and 18/22 EMR countries provided nominations: 46 interviews were conducted in the first stage and 18 in the second until data saturation was reached (Annex 1 in Supplementary material). The selection was based on the participant's role as policymakers, hospital managers, and/or members of senior management teams in hospitals treating COVID-19 across the 22 countries of the EMR. Participation was voluntary and, in most cases, KIs agreed to be interviewed only in a few cases, where the high workload and pressure of the pandemic responses, did they nominate other relevant stakeholders in their place. To optimize the diversity, comparability, and transferability of findings, no restrictions were placed on the type or size of the facility, participants represented 18/22 EMR countries, ranging from low, middle, and high-income countries, including countries in conflict settings and emergencies, and included various health cadres in management positions along with health professionals from various specializes.

KIIs were conducted online (using Zoom) for 50–90 mins by 2 members of the research team. Almost all interviews were conducted in English, with few conducted in Arabic, Persian, or French. In line with Lincoln and Guba's “naturalistic” criteria for qualitative research Trustworthiness, the four dimensions of credibility, dependability, transferability, and confirmability were considered to ameliorate the internal and external validity of findings (18). Active listening and probes along with prolonged engagement and immersion with the data were used to increase credibility and dependability. Following the repetition of themes during KIIs, the research team conducted a few additional interviews to confirm data saturation and reached a consensus. To improve confirmability and dependability, a record of analytical activities was kept. The interviews were audio-recorded and kept in secure files to be deleted within 2 years of project finalization. To improve credibility, the initial findings were shared with participants for discussion and feedback, the results were also presented in several regional webinars with key informants and technical experts, each with over 100 participants. The feedback was positive and did not significantly change the results.

Regarding the online surveys, upon revision and piloting, links were shared through two modalities: firstly, all key informants interviewed received a link to the survey (some of which confirmed to have responded while others shared within their networks), and secondly, through WHO country offices who disseminated the link to relevant stakeholders, including but not limited to Hospital managers, clinical directors, management teams, senior front-line health professionals, who were invited to participate and share the link within their respective networks. Surveys in both stages asked a few questions regarding participant demographics and hospital characteristics. The first survey was disseminated between July and October 2020 and was guided by the 10 domains of the WHO/EMRO hospital's COVID-19 readiness checklist. This survey included open-ended questions regarding hospitals' experiences, challenges, lessons learned, and the roles and expectations of hospitals, governments, and WHO in enabling recovery from COVID-19 which provided rich qualitative data for further analysis and triangulation. The second survey was disseminated between February and April 2022 and focused on evaluating hospital resilience by using Likert-scale questions, related to resilience to various types of hazards, responses and recovery from the last non-COVID emergency or disaster, changes to resilience capacities before and during COVID-19, and a checklist of available measurement tools, assessments, or evaluation strategies across six components for hospital resilience. This survey included open-ended questions on challenges/barriers (internal and external to the health facility) and practical tips/lessons learned through COVID-19 recovery on strengthening hospital resilience. To optimize responsivity, follow-up messages were sent regularly to remind participants to respond and widely share the survey.

### 2.2. Data analysis

Thematic (content) analysis was used following the six steps of the Braun and Clark approach (18, 19). Firstly, the research team transcribed the KIIs using electronic software and familiarized themselves with the data by reviewing, cross-referencing against the notes taken by the interviewers, and identifying initial codes. In non-English KIIs, a translation was made by the research team, and main notes were shared in English for summary, discussion, and consensus. Secondly, open coding was used and the research team systematically generated initial codes using an inductive approach. The MaxQDA software was used to organize and analyse all the qualitative data. Thirdly, two coders discussed the completeness of the data and reached a consensus regarding data saturation when no new concepts emerged. Fourthly, the coded segments were sorted to identify the main themes and sub-themes for the main research questions stated in the study objectives. Initial themes were organized and merged accordingly. Fifthly, the word-cloud functions of the software were used to generate the names of abstract themes and confirm the most cited ones. Finally, qualitative findings were synthesized, triangulated with survey results and literature review, and shared with experts for further validation (20).

As for the surveys, after data cleaning, a total of 139 survey responses were included from 14/22 EMR countries from the first survey, and 113 from 13/22 countries were included in the second. A descriptive analysis was also conducted using Microsoft Excel (Annex 2 in Supplementary material).

## 3. Results

For each of the four study objectives, qualitative findings captured the following themes and sub-themes detailed in the following section and summarized in Table 1.

**Table 1:** Themes and sub-themes by study objective.

**Study objective**	**Themes**	**Sub-themes**
***1. The role of hospitals in recovering from COVID-19***	***1.1. Education***	1) External/Community-facing: Rebuilding public trust, health promotion, and communication with the community, raising awareness, managing fear and misinformation, 2) Internal/Hospital-facing: Building capacities of frontliners
	***1.2. Risk reduction***	1) Infection prevention and control including managing visitors 2) Strengthening surveillance and information systems 3) Environmental impacts
	***1.3. Services continuity***	1) Utilizing telemedicine 2) Business/services continuity planning
***2. Hospitals' expectations of public health institutions*** ***to enable recovery from COVID-19***	***2.1. Hospitals' expectations of MOH***	1) Human resource management 2) Financial and logistical support 3) Leadership and management
	***2.2. Hospitals' expectations of WHO***	1) Source of evidence-based information 2) Coordination 3) Resources mobilization
***3. Evaluation of hospital resilience before and*** ***through COVID-19***	***3.1. Resilience to various types of hazards***	Per WHO hazards categorization: Natural, Biological, Technological, Societal, and Environmental
	***3.2. Resilience capacities across DRM stages***	1) Resilience to the last non-COVID emergency or disaster 2) Changes in hospital resilience before COVID-19 and now
***4. Lessons to strengthen hospital resilience throughout*** ***the COVID-19 recovery***	***4.1. Resilient staff***	1) Availability and mobility, 2) Competencies and in-service training, and 3) Physical, mental, and financial safety
	***4.2. Sustainable finance***	1) Back-up funding for emergencies, 2) Financial literacy of hospital managers to ensure informed decision-making, and 3) Diversity income sources
	***4.3. Adaptive leadership and management***	1) Learning and adapting strategies and systems, 2) Hospital-level preparedness and response programs, and 3) Empowering frontline stakeholders (including the community) to ensure swift decision-making

### 3.1. Role of hospitals in recovering from COVID-19

**Figure 1:**
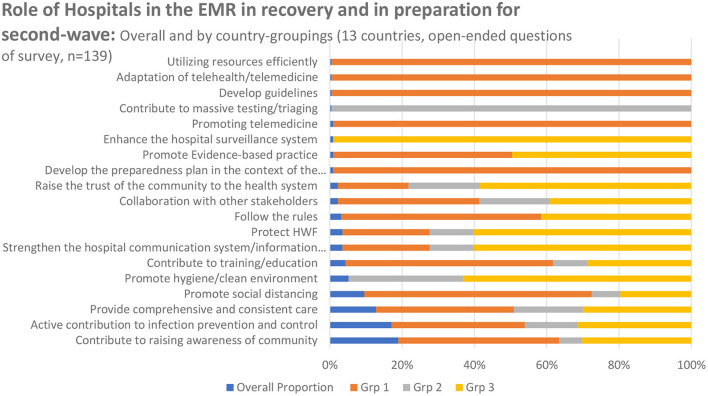
Survey responses ranking role of hospitals, in the EMR, in recovering from COVID-19; by country group and overall.

**Figure 2:**
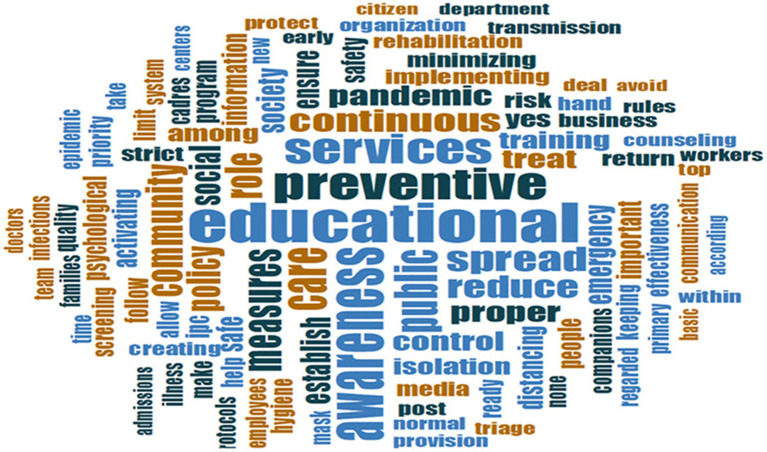
Word cloud of qualitative responses on role of hospitals in recovery from COVID-19.

Following the first wave of COVID-19, hospital managers, and frontline workers reflected those hospitals have a major role to play not only in the initial response to the pandemic, but also in the recovery, transition to “normalcy,” and preparation for forthcoming surges. The most common themes included: (1) **education** (including health promotion and communication to raise awareness in the community and strengthening capacities of frontline staff), (2) **risk reduction** (including infection **prevention** and control), and (3) **service continuity** (Table 1). These themes were reflected as the top three interventions across survey respondents (Figure 1) and further confirmed by Figure 2 whereby the most frequently mentioned concepts and words are largest in font, including: “educational,” “awareness,” “preventive,” “services,” “continuous” and “care.”

#### 3.1.1. Education

Education was the most prominent theme across the qualitative findings (Figure 2). During emergencies, hospitals have an external-facing obligation toward educating their patients and communities, working together with other actors and partners within a whole-of-society approach, but also have an internal-facing obligation to train and capacitate their staff (Table 1). The role of hospitals to educate their health workers will be addressed under Section Resilient staff.

The vast majority of survey respondents noted that hospitals must play a role in the recovery phase by being a strong force for health promotion (education) and raising awareness about preventive and public health measures to the general public particularly during emergencies (Figures 1, 2). Hospitals have an essential role to play not only in service delivery but more significantly in rebuilding the public's trust in the health system through health promotion and health education. Emphasis was placed on providing “reliable information” and “not spreading false rumors to intimidate society.” Healthcare providers and frontline workers advised using “social media,” “brochures” and technology to spread awareness and educate the public on social distancing, isolation of suspected and confirmed cases, and handwashing as the most important IPC strategies for everyday life and return to work. Hospital managers reflected on the impact of mobilizing and collaborating with the community not only in rebuilding trust but also in resuming hospital operations:

***“As part of our communication efforts: (1) our staff educated patients and their families to avoid visiting the hospital unnecessarily, (2) we arranged awareness sessions in our colleges and education institutions to empower the youth regarding vaccination and personal protective equipment, and (3) we mobilized our community to help the hospital” (KI 1)***.

Raising awareness in the community to actively contribute to the reduction of infections was a key role of hospitals in the recovery. According to respondents, this directly resulted in minimizing public health and economic threats enabling society to return to normal after the pandemic.

#### 3.1.2. Risk reduction

Participants highlighted the essential role that hospitals and health facilities play in fulfilling the health system's public health functions, whether in health promotion and education; risk reduction and IPC; or surveillance.

One issue that was raised extensively among key informants and survey respondents alike was the management of visitors which posed a threat to cross-infections (Table 2). Notably, hospital managers learned and adapted to ensure safety and high-quality both patients and staff, many mentioning the shift in infections from nosocomial during the first wave to community-based during the subsequent waves. They also stressed the importance of reducing the risk of cross-infections between COVID-19 and non-COVID patients to ensure the continuity of essential health services and limit disruptions to operations (Table 2).

**Table 2:** Priority themes among different types of health professionals regarding the role of hospitals in recovery from COVID-19.

**Management: Head of Deparment/Directors/Policymakers**	**Nurses**	**Physicians**
“*Regular and more education for the public on social media and television regarding the transmission of infection and safety precautions. This will reduce fears and myths and at the same time increase awareness in the public to continue taking all safety precautions”* “*It is easy to return to the pre-pandemic business mode with an emphasis on infection control”* “*Hospitals should be and remain a safe place for patients. We must work to provide services for both COVID patients and other patients smoothly and safely as possible“*	“*We have to pass this hard time by having awareness of infection control and act as role models in preventing infection”* “*The hospital administration is trying very hard to reduce and limit the spread of the disease. The issue remains a ‘cultural issue' in individuals and the community”*	“*Hospitals should collaborate with the community to win the battle”* “*Balancing resumption of services with the safety of patients and staff”* “*Constant awareness of hospital visitors. Spreading educational brochures among cadres and visitors”* “*Disseminating awareness videos among the community through various means of communication”*
“*Supporting doctors and creating medical and psychological assistance teams”*	“*Psychological health education is the most important factor”*	“*Establish a business continuity plan that allows the department to run its emergency plans during COVID-19”*

Additionally, some respondents suggested that hospitals should be involved in “widespread surveillance; with ongoing data collection,” should utilize “robust screening and triage practices,” and should ensure “early detection and reducing spread of disease.” On the other hand, few respondents noted the hospital's role in reducing risks more broadly related to environmental sustainability, suggesting the need for “more rational utilization of resources, such as consumables, personal protective equipment [PPE], and basic medical supplies” and “minimizing wastage at hospital level.”

#### 3.1.3. Services continuity

About a quarter of participants described the role of the hospital as primarily to “treat the illness,” and provide “quality care and clinical management of COVID-19 cases.” Nevertheless, hospital managers across the Region highlighted the use of telemedicine to reduce the burdens on the hospitals and the need for service (and business) continuity planning including efficient coordination and management of limited human, financial, and material resources for surges (Table 1).

A sub-analysis among different types of frontline workers revealed a general agreement between professional groups regarding the importance of health promotion/education and increasing awareness in the community (including health workers) to ensure safety in service provision. Physicians emphasized the need to work collaboratively with the community, considering them as a partner in the pandemic response. Nurses and administrators highlighted the need for a culture change, both within hospitals and the community, regarding the perceptions and practices of IPC. Health workers expressed different priorities when it came to planning for services continuity in the recovery (Table 2). Nurses and administrators alike emphasized the need for staff mental health and psychosocial support. On the other hand, physicians highlighted the need for business continuity plans to ensure that future emergencies or surges don't disrupt the provision of care.

Across the EMR, the role of hospitals in the recovery stage can be summarized through the three overarching themes mentioned above. While all three themes were commonly mentioned across countries of all-income groups (Figure 1); their operationalization varied depending on resources. For instance, the second theme regarding infection prevention and control (IPC) and reducing risks is interpreted differently between high and low-income countries. In most high-income countries (Grp 1), respondents indicated that the role of hospitals in the recovery is to “follow the rules,” “develop guidelines according to national strategy,” “contribute to training,” “promote social distancing,” and reduce the load on hospitals through “use of telemedicine.” On the other hand, in lower-income countries (Grp 2 and 3), preventing infections and reducing risk looked like “enhancing hospital surveillance and information systems,” “rebuilding the trust of the community in the health system,” “protecting health workers” and “promote a hygienic environment.” The latter reflects the need for overall health system strengthening and stabilization, particularly in countries facing ongoing humanitarian crises. Notably, across both the highest and lowest income country groups equally, respondents reflected the need to “promote evidence-based practices (EBP)”; however, the implementation of these EBP is directly related to hospital culture which is influenced by numerous factors. Managers reflected on the challenges of the nuances in hospital culture, the interplay of society and community, and the perceptions of health workers (as community members) as factors that must be considered to uphold IPC, combat stigma, and resume health services. Furthermore, stakeholders from the Region, across countries of all-income groups, highlighted the need for creating a safe and supportive working environment and reducing occupational risks and deaths, especially in the early months of the pandemic.

### 3.2. Hospitals' expectations of public health institutions to enable recovery from COVID-19

For this study, we asked hospital managers their expectations of their ministries of health (Section 3.2.1: Hospitals' expectations of MOH) and of the WHO (Section 3.2.2: Hospitals' expectations of WHO) in enabling recovery from COVID-19; the sub-themes were generally similar, particularly in the Region's resource-retrained settings (Table 1).

#### 3.2.1. Hospitals' expectations of MOH

**Figure 3:**
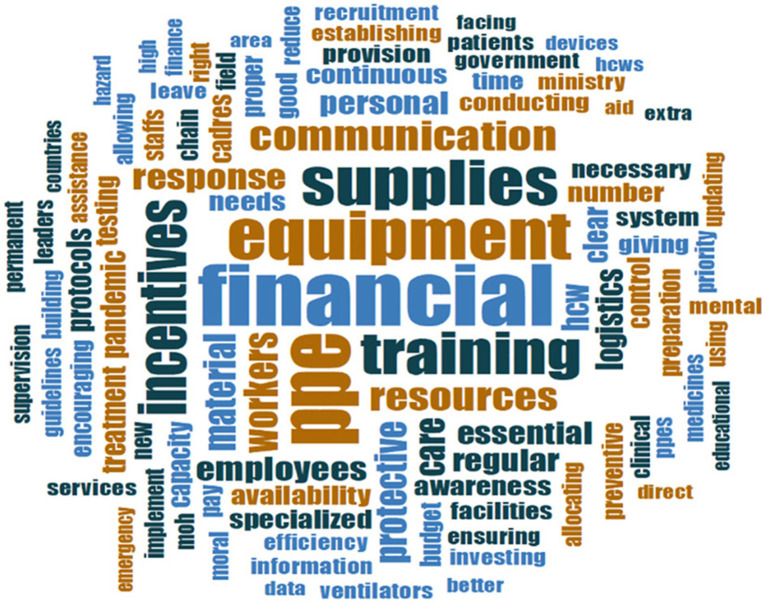
Word cloud of qualitative responses on hospitals expectations from MOH in enabling recovery from COVID-19.

Qualitative findings revealed that the four major requests from their respective national MOH were related to **(1) human resource management (HRM), particularly remuneration, training, increased staffing, and psychological support, (2) financial and logistical support, (3) leadership and management (including communication and clinical support)** (Table 1). Table 3 highlights the main sub-themes (including sub-sub-themes and examples), raised by respondents regarding the expectations of MOHs in supporting hospitals directly responding to COVID-19 which are further reflected in Figure 3 through the prominent words “financial,” “incentives,” “equipment,” “supplies,” “PPE,” “training” and “communication.”

**Table 3:** Most frequent themes regarding how the MOH could support hospitals responding and recovering from COVID-19, in order of frequency.

**Sub-theme**	**Sub-sub-theme**	**Examples**
**Human resources management (HRM)**	Incentives	“*Providing incentives for workers,” “Implement incentives system for staff,” “Give money to staff,” “Pay incentives for staff on time, and regularly,” “Give graduated students scholarships and grants for qualification,” “With material incentives for worker,” and “Providing incentives/ hazard pay”*
	Training	“*Training of cadres,” “Train health workers,” “Helping in queuing training,” “Regular training of staff,” “Training on IPC,” “Trained personal are essential and worth investing in, having infrastructure for capacity building,” and “Qualify all staff for an anticipated emergency even those who are in primary health care and psychiatric hospital”*
	Staff number specialization	“*Supporting the hospital with human resources,” “Recruitment of extra staff,” “Provide the number of employees,” “More staff recruitment to avoid overburden,” “Reduce the work load of staffs,” “High staff,” and “Putting the right employee in the right job arrangement of paramedical staff”*
	Psychological support	“*Support the staff, listen to their concerns/allow the staff to verbalize their feelings because it's really difficult for them to handle this situation because of fear of contracting COVID-19,” “Frequently test and vaccination of staff,” “stress management,” “Be sure for the physical and mental wellbeing of staff,” “Counseling, emotional support,” “Allowing employees to take leave to rest because it is one of their rights,” and “To provide more psychological support, Support how, “Moral support”*
**Financial and logistical support**	Support and incentives	“*Financial aid, financial support,” “Providing satisfying financial support” “Financial compensation for the staff,” “Support is in financial resources,” “Provide the budget for the financial health facility completely,” “Motivating medical personnel financially enough to motivate them to work,” and “Supporting health workers and strengthening them financially and psychologically”*
	PPE	“*Full PPE support,” “Prepare PPE,” “Providing quality PPE,” “By providing enough PPE for healthcare workers,” “To provide enough PPE kit to all staffs,” and “Provide for the needs of the hospitals, especially for PPE”*
	Other equipment	“*Good equipment,” “Hospitals affording material aid and equipment,” “Allocate hospitals in each specialty area for a respiratory infection that is equipped with equipment,” “Providing all medical equipment and supplies, and medicines,” “Providing devices and equipment that we lack in isolation centers, such as ventilators,” and “To provide the essential material, supplies, and equipment”*
	Supply chain management	“*Efficiency in supply chain Management,” “Organization of adequate medical supplies,” “Providing supplies, devices, and medicines and ensuring their continuous flow,” “Fast supply chain,” “The regular provision of medical and non-medical supplies,” “Keep supply chain maintained,” “Maintain the supply of essential items,” and “Clear communication pathway-unified supply chain”*
**Leadership and management**	Communication	“*Good communication,” “Stop mixed messages,” “Communication of government leaders with HCW and encouragement through field visits,” “Involvement of the stakeholders,” “Clear with employers,” “Be transparent,” “MOH regularly meets with frontline leaders from hospitals,” and “Mass Communication”*
	Strategies	“*Commitment,” “Holistic administration of the pandemic,” “Effective communication system,” “Effective utilization of the resources central bed management,” “New management based organization on, performance and accountability, “Analysis of each hospital individually according to their need and respond to them,” “Professional rather political approach,” “Coordination and cooperation between the technical and administrative teams in crisis management,” “Situation analysis and review of outcomes,” “Data collection and transparent communication to HCW and the public,” “Encouraging, supporting, and conducting research, particularly in using off-label medications,” and “Sharing of resources”*
	Bed capacity	“*Allocate hospitals in each special area for respiratory infection,” “Central bed management,” “Arrange more beds for COVID-19 pts,” “Sufficient specialized ward with adequate medical items,” “Create new secondary care hospital so care continues their regular services”*
	Guidelines	“*Case definitions,” “Management protocols,” “Centralized guidelines, institution rather than individualized protocols,” “Update the local policy and share it with the end users,” “Support by updating recommendations and strategies relevant to the various target populations of COVID,” and “Enforced regulations and SOPs”*
	Diagnostic capacity	“*Activate the work of laboratories by securing and controlling materials and kits necessary for laboratory work,” “Government to provide adequate diagnosis and treatment facilities,” “PCR testing,” “Continue tests even from outside of the hospital,” and “Early detection”*
	Medical treatment	“*Try to find proper vaccines,” “Providing effective treatment,” and “Free treatment”*
	Research	“*By calculating the no of recoveries,” “Data collection and transparent communication to HCW and public,” and “Encouraging, supporting and conducting research, particularly in using off label medications”*

In the early phase of COVID-19 response and recovery, qualitative findings identified that hospitals expected better human resource management particularly regarding financial and logistical support (whether through fixed contracts, more secure remuneration, improved incentives, or provision of sufficient PPEs, supplies, and equipment), as well as training. Respondents raised that more holistic incentive packages may encourage staff to work with COVID-19 despite significant fears of occupational infections and significant illness, they may also encourage clinicians from other disciplines or remote locations to volunteer their help when the healthcare system is overburdened. Additionally, high-quality clinical care requires adequate staff numbers as well as a reliable supply chain for PPE, diagnostic services, oxygen, medical equipment, and medication-survey respondents felt that the MOH has an important role to play in providing and ensuring the ongoing availability of these materials (Figure 3; Table 3). Moreso, survey results indicated that among the most frequently cited hospital requests to MOH were around the themes of logistical, financial, and managerial support, including providing adequate medical supplies, equipment, and PPEs (around 22%), securing adequate qualified critical care staff and specialists (15%), and increasing financial support (about 12%).

When exploring a sub-analysis by types of health professionals, all hospital staff in clinical and managerial roles including heads of clinical departments including nursing, senior management teams, physicians, nurses, and IPC specialists, found logistical support and the provision of supplies, equipment, and PPE chief among expectations of MOH. Clinical staff, namely doctors and nurses, identified financial support in the form of incentives as the main request from their governments. Regarding HRM and the distribution of the health workforce, hospital directors, members of senior management teams, and nurses expected MOHs to secure sufficient and adequately trained numbers of specialists across designated hospitals responding to COVID-19. Hospital managers complained that the shortages of specialists posed a major threat to the response, especially in resource-restrained countries in the Region where workforce shortages and maldistributions are common. Both clinical staff (physicians and nurses) and members of the senior management team highlighted the need for increased staff mental health training, psychosocial support, recognition, and efforts to raise health worker profiles and morale; these were considered top expectations of MOH in the early months of the pandemic.

Generally, hospitals in the EMR's high-income countries are more likely to anticipate ministerial support in promoting telemedicine, raising awareness in the community, and enforcing measures related to social distancing, compulsory mask use, screening, and testing. On the other hand, hospitals in the Region's LMICs expected governmental support expected stronger leadership, smoother coordination of actors (including the private sector), efficient HR management, increased training, as well as financial and logistical support, whether through fixed contracts, more secure remuneration, improved incentives, or provision of sufficient PPEs, supplies, and equipment. Notably, in most Group 3 countries, hospitals are more likely to request a wide range of technical, financial, managerial, and logistical support from ministries and government, WHO, and other international organizations. This was attributed by respondents to the fact that their hospitals were facing COVID-19 as well as other humanitarian emergencies, within fragile health systems further strained by the public health and financial pressures exacerbated by the pandemic and ongoing conflicts.

In addition to the primary obligation of MOH to disseminate clinical guidelines to hospitals and update them according to international standards, respondents expected ministries to improve their **leadership and coordination** (Table 3). Hospitals expected clear communication and early involvement of multiple stakeholders to ensure a unified response. Another issue raised in the first phase of our study was the burden on hospitals to provide different sets of data and information in different modalities/platforms to various directorates in their MOHs; hospital managers expected to have a more integrated approach toward information management at the central level. One of the top issues expected of the government was proactive preparedness and more comprehensive contingency planning related to all aspects of the health system, including early procurement of supplies and equipment such as PPEs, medicines, and testing kits. This was especially highlighted across hospitals in resource-restrained and conflict-affected settings. Further to this, hospitals expected greater coordination in the form of referral pathways, and distribution of ICU beds, ventilators, medicines, medical supplies, and equipment.

#### 3.2.2. Hospitals' expectations of WHO

Regarding the role of WHO in supporting hospitals' responses to COVID-19 in the early months of response and recovery, hospital managers expected several key interventions (Table 4) including but not limited to providing technical support and guidelines, raising awareness and keeping the public, implementers, and frontliners updated on latest evidence-based practices, building capacity (including technical and managerial capacities), coordinating between health actors and ensuring financial and logistical support through adequate resource mobilization, especially in resource-restrained and humanitarian settings, and finally in research and development (particularly related to vaccine development and distribution). These interventions can be synthesized into three main sub-themes: **(1) WHO as a reliable source of evidence-based information, (2) WHO as a politically-neutral actor in coordination, and (3) WHO as a support in resource mobilization**.

**Table 4:** Most frequent sub-themes related to how the WHO could support hospitals responding and recovering from COVID-19, in order of frequency.

**Sub-theme**	**Sub-sub-theme**	**Examples**
**Source of evidence-based information**	Technical guidelines and training	“*Prepare guidelines conduct training”*
		“*Guidelines, safety at work place and home, public awareness”*
		“*Sharing appropriate treatment protocols and guidelines training”*
		“*Staff how to handle pandemic (disaster plan)”*
		“*Regular guidelines”*
		“*By providing proper guidelines in advance!”*
		“*Provide us with EB guidelines, success stories from other countries”*
		“*Disseminate information in real-time, the establishment of Protocols and guidelines”*
		“*Guidance and counseling”*
		“*Guide lines for health care workers safety”*
		“*Continuous training and qualification for health staff and continuous medical guidance”*
		“*By supporting new hospital strategy conceptualization of new models—training—expertise”*
	Evidence and research	“*WHO should be independently evaluating the data on certain treatments/ interventions—not influenced by social media, countries or public or politics”*
		“*Provide us with EB guidelines”*
		“*Scientific update”*
		“*Revised protocols”*
		“*Real identification of the elements that work scientifically and practically and communicating with them with the COVID epidemic”*
	Essential services	“*Aid to continue providing basic services during the emergency period in order to limit the number of direct or indirect death and”*
		“*Ensure the continuation of providing the necessary services to the citizens until they obtain the necessary support from medicines, equipment, and consumables essential for work”*
	Innovation	“*New invention of preventive measures”*
		“*Updates to vaccine and treatment”*
**Coordination**	Direction and accountability	“*Universal policy for All hospitals under the ministry of health in combatting COVID-19”*
		“*Ensuring the Ministry's commitment to implementing rehabilitation projects for health institutions and supporting health institutions far from the center”*
		“*Direct supervision and evaluation”*
		“*Cooperation, equality and justice”*
		“*Good coordination”*
		“*Set clear policies, oblige the Ministry of Health to establish quality and infection control department”*
		“*By visiting some of the hospitals randomly and acting realistically”*
		“*Classifying countries according to severity”*
		“*To listen”*
		“*Vigilance and support,” “Prioritize and guide the allocation and targeting of resources to achieve the goals”*
**Resource mobilization**	PPE, medical	“*Hospitals affording material aid and equipment”*
		“*Personnel and protective equipment”*
		“*Providing devices and equipment that we lack in isolation centers”*
		“*Ventilators”*
		“*Medical equipment support”*
		“*Oxygen insurance and protective equipment”*
	Finances	“*Support financially”*
		“*Assistance with the operational budget, such as patients' meals and other supplies”*
		“*Provide scholarships and grants for staff”*
		“*Try to stimulate the staff through incentive support”*

In most high-income countries, hospitals highlighted the essential role of WHO in providing technical and informational support, in addition to this, hospitals in lower-and-middle-income countries, especially those in humanitarian settings, also rely on WHO for resource mobilization through financial and material support. Across all countries, the role of WHO was highlighted in building the managerial capacities of hospital directors.

##### 3.2.2.1. WHO as a reliable source of evidence-based information

**Figure 4:**
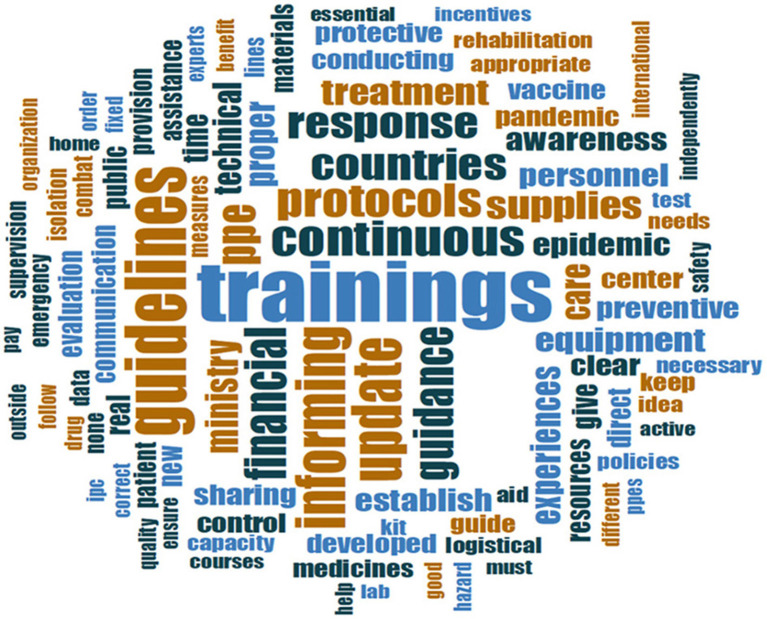
Word cloud of qualitative responses on hospitals expectations from WHO in enabling recovery from COVID-19.

Among survey respondents, the most significant theme regarding the role of WHO in supporting hospitals responding to and recovering from COVID-19 was providing “evidence-based information” (Table 4; Figure 4). Based on participants' responses, this encompasses: (1) “technical guidelines,” (2) “capacity building and training,” (3) “technical support to recover and continue essential health services,” and (4) “innovation for rapid and safe vaccination.”

Firstly, hospital managers throughout the Region relied on WHO to provide technical guidance, not only related to the nature of the virus, its epidemiology, infectivity, and transmission but the implications on hospital management and clinical practice. The onset of a new and evolving virus brought heightened anxiety due to the limited evidence and widespread misinformation. Hospitals in the EMR expected WHO to continuously provide and update reliable evidence-based guidelines and recommendations, guide clinical management, implement IPC protocols, ensure hospital operations run efficiently and safely, and increase preparedness and resilience for surges, especially in the early stages of COVID-19 response and recovery. Hospital managers and health workers considered WHO a trusted entity to verify and disseminate reliable and updated evidence regarding IPC, emergency and surge preparedness and response, and clinical management (including identification, diagnosis, and treatment) of COVID-19. One hospital manager mentioned:

***“[WHO] is the most important job all over the world, just to understand this new disease and update the critical protocols. For instance, I expect WHO to have a clear understanding of the duration of infectivity; if we should decrease isolation from 10 days to 9, it has a huge impact on the hospitals” (KI 2L)***.

Secondly, hospitals expected capacity-building support from WHO, as confirmed by the two largest words (most frequent themes) of “training” and “guidelines” (Figure 4). In the first quarter following the announcement of COVID-19 as a Public Health Emergency of International Concern (PHEIC) (July 2020), training, health workforce strengthening, and the use of telemedicine (including its use for education) have been identified among the top three requests across two-thirds of the Region. Hospital managers in resource-restrained and humanitarian settings, where critical shortages of health workers and specialists are chronic health systems stressors, confirmed the need for continuous education and in-service training and re-training to ensure that frontliners (including students, volunteers, and health workers from various specialties) have adequate competencies to provide critical and emergency care safely.

Thirdly, hospital managers expected WHO's technical expertise to support countries in maintaining and monitoring essential health services and transitioning health systems back to normalcy and recovery. In the early months of response, one hospital manager mentioned:

***“Nobody's talking about the recovery phase yet because everybody's talking about the second wave. A second wave is a concern, but people are not only dying from COVID, but they will start dying from us not providing health care. We need to know how to recover safely” (KI 3)***.

Hospital managers across the EMR relied on WHO to build capacities in emergency and disaster preparedness, leadership, supply chain management (especially in FCS), risk communication and health promotion, HRM, mental health and psychosocial support for front-liners, clinical management (including triage, screening, diagnosing, strengthening laboratory capacities, providing critical care, managing COVID-19 co-morbidities, treatment in isolation wards, etc…), maintenance and expansion of essential health services, use telemedicine, and improvements to hospitals' quality, safety, and IPC measures. In the face of critical staff shortages, high workload, and burnout, hospitals in resource-restrained and humanitarian settings promptly identified the need to build the capacities of clinicians on stress management and greater emphasis on mental health in crises and psychosocial support:

***“Beyond training staff in isolation centers and clinical areas, there is no focus on psychosocial support from WHO or any other organizations” (KI 4)***.

Fourthly, hospital managers expressed that WHO had a timely responsibility to support ongoing research, evidence generation, knowledge sharing, and dissemination, as well as documenting and evaluating innovative interventions in triage, treatment, and vaccine development. Though there are vast inequalities in access to COVID-19 vaccines globally, hospital managers attributed the development and distribution of vaccines as a critical enabler to early recovery from COVID-19. One hospital manager mentioned:

***“At the onset of the pandemic, there were no vaccination protocols yet. I secured them somewhere to be able to sleep at night and carry on their daily activities so that they won't be obliged to leave the hospital and expose their parents or their families to the transmission of COVID. The vaccines allowed us to resume almost-normal operations” (KI 5)***.

##### 3.2.2.2. WHO as a politically-neutral coordinator

Across the Region, especially in emergency and humanitarian settings, KIs highlighted the role of WHO as an objective actor and trustworthy source of reliable internationally sound standards and guidelines. In conflict-zones, where parallel governments may exist between opposing parties, hospital managers stressed the importance of WHO ability to remain apolitical and provide evidence to frontliners to deliver high-quality care:

***“The WHO is a reliable source of information and remains an honest broker. In our country, if guidelines are coming ministry of health of [x] region, they will not be followed, but they will agree if they are coming from WHO but not from the opposition” (KI 6)***.

##### 3.2.2.3. WHO as support in resource mobilization

In the early months of response and recovery, the top request from WHO was related to financial and material resource mobilization and timely procurement of essential supplies (namely PPEs). While some high-income countries, were utilized procurement channels through WHO; this was especially true in the Region's LMICs, particularly those health systems facing the double burdens of war/humanitarian conflicts and this pandemic. In many of these Region's emergency countries, WHO was responsible for the initial provision of PPEs, testing kits, medicines, supplies, and equipment. Hospital managers from at least 10 emergency countries also mentioned relying on the WHO to pay the salaries of frontliners in designated COVID-19 hospitals.

### 3.3. Evaluating hospital resilience before and through COVID-19

KIs qualitatively evaluated two dimensions of hospital resilience; firstly, hospital resilience to various types of hazards (according to WHO categorization), and secondly, through evaluating the hospital's resilience four capacities through DRM stages.

#### 3.3.1. Hospital resilience to various types of hazards

**Figure 5:**
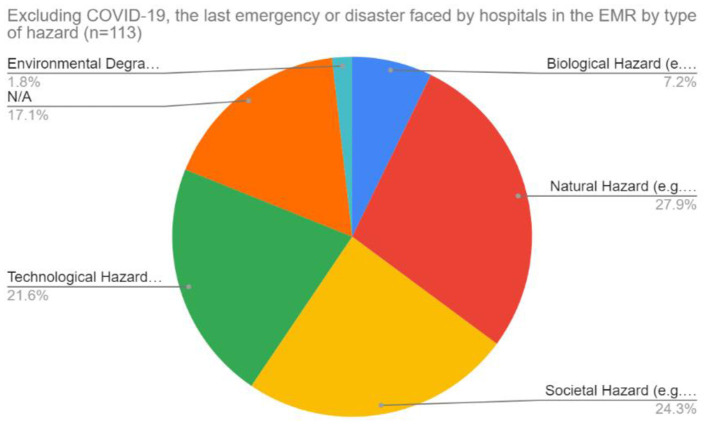
Last type of emergency or hazard faced by hospitals in the EMR excluding COVID-19.

Survey respondents were asked about the last non-COVID hazard facing their hospitals and then asked to evaluate their hospitals' resilience to the various types of hazards on a 5-point Likert scale from least resilient (1) to highly resilient (5). Apart from COVID-19, the most commonly reported type of hazards were natural (27.9%) and societal hazards (24.3%), followed by technological (21.6%), biological (7.2%), and environmental (1.8%) (Figure 5).

**Figure 6:**
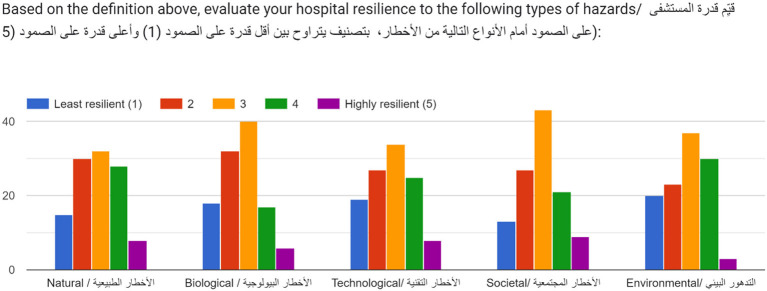
Five-point Likert-scale evaluation of hospital resilience by type of hazard.

Generally, hospital managers across the EMR neutrally evaluated their hospital's resilience to various types of hazards. All types of hazards, with the highest reported score was 3/5 (yellow) across all five categories of hazards (Natural, Biological, Technological, Societal, and Environmental) (Figure 6). Overall, findings revealed that the highest reported scores were across societal followed by biological hazards, indicating a medium or average level of hospital resilience to these types of hazards. With the exception of environmental hazards, findings reflect a skew toward “less resilient” with the second most frequent response in all graphs being 2/5 (Figure 6). Conversely, responses for environmental hazards indicate a positive skew toward “higher resilient” with the second highest response as 4/5. Across all hazard-categories, the lowest reported score was 5 (highly resilient—purple in Figure 6) indicating that most respondents did not perceive that their hospitals were highly resilient to any hazard. These scores further reflect the need to build on existing structures and efforts and improve hospital resilience to all types of hazards across the EMR.

#### 3.3.2. Hospital resilience through resilience capacities across DRM stages

**Figure 7:**
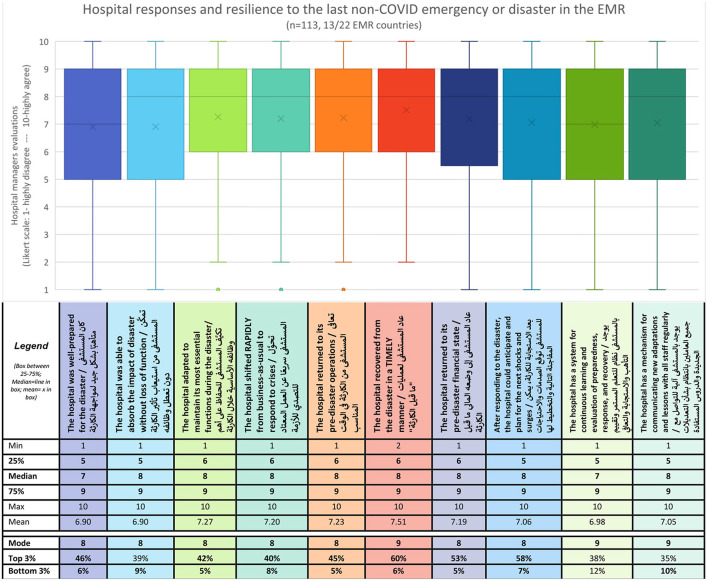
Ten-point Likert-scale evaluation of hospital resilience to the last non-COVID disaster or emergency.

With regards to hospitals' resilience before COVID-19, 10 statements were presented to respondents regarding hospital's responses to a non-COVID emergency or disaster, whereby respondents selected along a 10-point Likert scale where 1 corresponded to highly disagree and 10 to highly agree (Figure 7).

Generally, hospital managers and frontliners responded positively as indicated by mode scores for all 10 questions, where seven questions reported modes of 8/10 and three reported modes of 9/10 (Figure 7). This positive skew across all questions is further reflected, in the high median scores of 8/10 in eight of ten questions, a mean ranging from 6.9 to 7.5, and a small range where 50% of responses (between the first and third quartiles) were scored between 5 or 6 and 9 (Figure 7). Moreover, in seven of ten questions, more than 40% of respondents selected one of the three topmost scores (8, 9, or 10/10), with the other two questions just barely below 38 and 39% respectively. On the other hand, when exploring the three lowermost scores [Bottom 3 (%)], all but one question found that 10% or less of respondents selected these.

Notably, the question with the highest scores was related to *timely recovery (Q6)* with 60% of respondents scoring in the three topmost categories, resulting in a mean of 7.51. On the other hand, the question with the greatest variation in responses was related to *a system for continuous learning and evaluating preparedness, response, and recovery (Q9)*, where 12% of respondents selected the lowermost scores, and around 16% equally scored 5,7,8,9 and 10 (Figure 7). Similarly, a few graphs also reflected notable peaks around score 5 indicating neutral evaluations of *hospitals' ability to absorb the impact of disaster without loss of function* (Q2) and hospitals *having a mechanism for communicating new adaptations and lessons learned with all staff in a regular manner (Q10)*. These areas reflect opportunities for improving hospital resilience.

**Figure 8:**
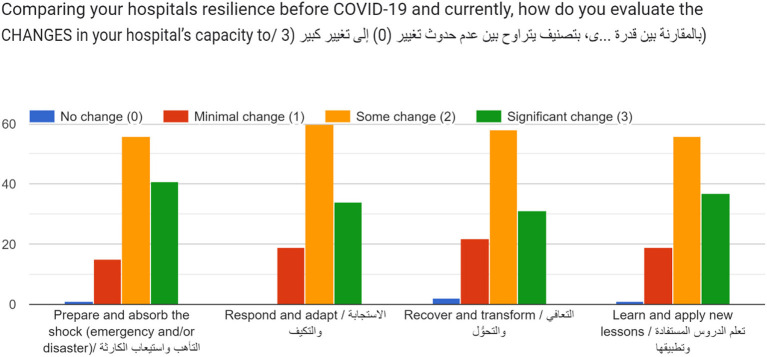
Evaluation of changes in hospital resilience before COVID-19 and in the current response.

To compare hospital resilience before COVID-19 and currently, an assumption was made to integrate and align the resilience capacities: absorb, adapt, transform, and learn, with the stages of DRM: prepare, respond, recover, and apply new lessons for prevention and risk mitigation. A question was posed over a four-point Likert scale ranging from No change (0) to Significant change (3). Generally, hospital managers positively evaluated the changes to their hospital's resilience capacities following the pandemic. Across all four capacities, hospital managers most frequently reported some change (2/3), with all four graphs positively skewed with significant change being the second most reported response across all capacities (Figure 8). Overall, hospital managers reported the most changes in their capacities to respond and adapt followed by their capacities to prepare and absorb shocks. Notably, although a total of four responses (4/113) were recorded indicating no change across all four capacities, two of these were related to the capacity to recover and transform. This capacity also recorded the highest score among minimal changes.

### 3.4. Lessons for strengthening hospital resilience through COVID-19 recovery

In response to asking hospital managers about their top lesson learned in strengthening their hospital resilience throughout recovering from COVID-19, the most prominent theme was related to strengthening hospitals' soft resilience through strengthening the resilience of the various hospital components with a particular focus on (1) resilient staff, (2) sustainable finance, and (3) adaptive leadership and management (Table 1).

#### 3.4.1. Resilient staff

The theme of health workforce resilience was among the most prominently mentioned across qualitative findings, and consistently with global and regional literature. The sub-themes include (1) availability and mobility, (2) competencies and in-service training, and (3) physical, mental, and financial safety (Table 1).

In the early response, hospital managers reassigned staff from other departments including specialists in primary care, emergency medicine, critical care, respiratory, cardiology, and internal medicine specialists to ICUs. In the face of critical shortages, KIs further reflected task-shifting volunteers, retirees, students, and residents to support in the COVID-19 response; this was especially necessary for the early stages when health workers were getting infected and needed to quarantine and recover over weeks. Relatedly, hospital managers noted the need for cross-training staff on all emergency and disaster response and management initiatives and activities to ensure adequate competencies:

***“Our staff cannot just be highly specialized in one area, we need to ensure that they have at least the needed basic skills to be mobilized or redeployed where needed” (KI 7)***.

***“During the pandemic, what was very important is cross-train the staff, the skill-mix training. We can't train a nurse [from scratch] during a crisis. So, mobility of human resources between units is also essential” (KI 8)***.

Moreso, hospital managers across the Region reflected on continuous training and learning as a key enabling factor to resilience. To improve the timely transfer of new information and knowledge, hospital managers noted utilizing e-learning, social media platforms such as WhatsApp, and intensive hands-on in-service training for frontliners. KIs further reflected on the importance of continuous improvement and creating a culture of learning at the facility-level as a core pillar to recovery and ultimately resilience.

Hospital managers further reflected on the various interventions used to protect and sustain “their most valuable resource” with particular emphasis on protecting health workers' wellbeing, especially during the prolonged and intertwined response and recovery phases of COVID-19:

***“Our first priority was to keep our human resources safe from any harm, we distributed PPEs daily. We implemented the guidelines issued by the infection control department. We arranged training led by the infection control department and medical directorates via zoom for all staff: faculty, nursing, paramedics, and even staff working in non-clinical areas” (KI 1)***.

Across the EMR, hospital managers from Afghanistan, Iran, Lebanon, Pakistan, Palestine, Oman, and Saudi Arabia, mentioned the motivation of human resources as a key enabler of hospital resilience. One hospital manager mentioned:

“***The personnel should be really satisfied to work in a hospital, not obliged to. The dedication of the staff to helping people or their loyalty to the hospital in which they are working. This is the cornerstone strength that this hospital had which allowed us to open and respond to COVID-19” (KI 5)***.

Across the Region and throughout the last 3 years of COVID-19, hospital managers attributed their hospital's resilience to the courage, humanitarian spirit, commitment, and sacrifices made by health workers, especially in some LMICs where their remuneration was often delayed and inconsistent due to national financial and political crises. Despite the difficult financial crises, one of the major interventions highlighted by hospital managers in LMICs and FCS was securing their health workers' timely compensation:

***“We did not furlough, we did not terminate, we did not fire people, but we reviewed compensation methodology to ensure that we are able to pay our employees what they deserve and motivate them during the crisis” (KI 8)***.

#### 3.4.2. Sustainable finance

In many of the Region's LMICs, one of the most critical issues raised related to hospitals' early recovery and ultimately resilience was finance and its implications on staffing, logistics, and supplies. Challenges were especially exacerbated in countries with political instability exacerbated financial crises and fragmented procurement which affected hospital operations as reflected by hospital managers from Afghanistan, Lebanon, Pakistan, Somalia, and Sudan. One key informant shared: ***“Financing played a key role in adaptability. The restrictions of cash flow in the country following the crisis affected the continuity of services” (KI 9)*** while another hospital manager reflected on the harsh economic crises over the phases of COVID-19 response: ***“In the first wave, the dollar was 1,500 pounds, now every dollar is 25,000 pounds. In face of this big inflation and the high cost of the maintenance contracts for repairing the damages, we are facing an economic crisis not only a health one” (KI 5)***.

KIs, especially those in resource-restrained contexts, recommended that every hospital manager should have back-up funding for emergencies which can be immediately mobilized during a crisis. One hospital manager stated: ***“The administrator must always have a financial reserve in the budgeting dedicated and put aside for extraordinary pressure for extreme cases that a hospital might face” (KI 5)***. KIs also stressed the need for hospital managers to be financially literate and have a pragmatic understanding of financial analyses of budgets along with a committed knowledgeable team to inform staffing and procurement of supplies:

***“To improve hospital resilience, the manager must have internal finance and administrative systems, detailing the income, contributions from which departments and number and skill-mix of health workers” (KI 10)***.

Further to this, hospital managers in LMICs and countries in emergencies also noted the need for financial autonomy with clear accountability mechanisms, diversifying hospital income sources, conducting internal audits to cut unnecessary expenditures, and doing medium-term and scenario planning based on various revenue streams. One hospital manager reflected:

***“How does the institution make itself financially sustainable or financially resilient? The solution is multifold starting with a diversification of income sources, because if the institution is only dependent on income from the hospital, then it will take a very big time to recover” (KI 8)***.

Despite the diversities in finance management systems across the Region, in many EMR countries in emergencies, hospital managers urged for an increase in hospital budgets allocated by the government as well as increased autonomy to expedite (financial, material, or human) resource mobilization.

#### 3.4.3. Adaptive leadership and management

The theme of adaptive leadership and management was highlighted by hospital managers who reflected the importance of the “systems” components, particularly the continuous improvement of strategies and processes throughout the ongoing response and recovery cycles. The subthemes include: (1) learning and adapting strategies and systems, (2) hospital-level preparedness and response programs, and (3) empowering frontline stakeholders (including the community) to ensure swift decision-making (Table 1).

Firstly, the constant adaptation in guidelines, communication of changing protocols, information, and knowledge sharing intra and inter-hospitals, and between hospitals and ministries of health, was among the most critical enablers to hospitals' resilience in the EMR. To improve hospital resilience, hospital managers reflected on the need to learn and adapt their strategies to improve service delivery. In the face of COVID-19, hospitals integrated telemedicine and embraced technology to improve their operations. One hospital manager from Lebanon further cited the hospital's use of artificial intelligence to identify available COVID-19 beds between the hospitals of the governorate while another hospital manager from Pakistan highlighted the use of zoom to provide ICU and IPC training to surrounding regional and provincial hospital staff. Nevertheless, hospital managers further reflected the need for an established system to enable this systematic or rapid adaptation of protocols and strategies: ***“Take the good learnings from the pandemic and apply it on a day-to-day basis; that is going to be the way forward” (KI 4)***.

Secondly, hospital managers reflected that while adaptation in crisis is necessary and predicted, there needs to be a system in place to enable learning, adaptation, and transformation, ultimately enabling resilience: “***When something happens, you don't have time for hospitals to adapt, there needs to be a process and a system in place as to how does one deal with the crisis and what is required to be done” (KI 8)***. Further to this, KIs reflected on the importance of proactive preparedness based on risk assessment and risk prioritization, in line with national DRM efforts. Moreso, HMs highlighted the need for hospital-level multi-hazards emergency preparedness and response programs, plans, and strategies, which ideally include all hazard and risk-informed contingency plans, service and business continuity plans, and recovery plans with a build-back better approach. HMs also stressed the importance of establishing a multidisciplinary hospital incident command system with clear communication and assigned roles and responsibilities to act in unity and speed and enabler adaptive management:

***“The [most important] part of disaster management is preparedness: You have time for risk assessment and to develop some emergency operation plans, you have time to improve your capacities, to develop early warning system, educate and train staff and use simulation exercises and drills, engage and communicate with the community, and finally learn from after action reviews and corrections. This will help you improve your resiliency” (KI 11)***.

Thirdly, hospital managers across the EMR frequently mentioned “agile and adaptive management” and “swift decision-making” as enablers of hospital resilience. Within the hospital, senior managers worked to empower middle management to improve processes and strategies and shifted the decision-making autonomy closer to the implementation and impact. Across the Region, hospital managers identified a gap in training and the need to build the competencies of hospital managers in DRM. Furthermore, decentralizing decision-making power to ensure swift action was a principal lesson highlighted by hospital managers across high-income and resource-restrained countries:

***“Resilience is transferred from top to bottom” (KI 10)***.

***“We had to move away from the traditional bureaucratic decision-making procedures; we were able to do so much during the pandemic, just because we were taking quick decisions” (KI 8)***.

***“During the crisis, if [staff] don't have that space of authority (autonomy to make decisions), then they are not likely to be resilient. Initially, all the decisions that came from leadership were cascaded down. Today, our front staff and our middle management are actually making decisions and improving whatever is needed to meet this demand without actually waiting for senior leadership” (KI 7)***.

## 4. Discussion

This study sought to address a prominent research gap in hospital resilience, especially through the recovery stage. Based on the reflections of hospital managers and frontline workers from combatting COVID-19 in the EMR, this qualitative paper explored four main questions: (1) the role of hospitals in recovering from COVID-19, (2) Hospitals' expectations from their public health institutions to enable recovery from COVID-19, (3) Hospital managers' evaluation of their hospitals' resilience before and through COVID-19, and (4) their lessons to strengthen hospital resilience throughout the COVID-19 recovery.

Firstly, according to frontliners, the role of hospitals in recovering from the pandemic includes health education, risk reduction and prevention of infections, and service continuity. In the first quarter following the declaration of COVID-19 as a PHEIC, hospital managers and frontline workers in the EMR concluded that hospitals have a critical role in recovering from the pandemic, not only in the early recovery stage but throughout the prolonged response in returning operations to “normal.” This proved to be true, 3 years later, as the world continues to combat COVID-19 and prepare for subsequent surges manifested by different variants. Frontliners aptly noted the hospital's role in fulfilling their primary functions in service delivery but also additional functions in health promotion, community engagement, and risk mitigation. This is consistent with global literature on hospitals during health emergencies where the primary objective of resilient hospitals is to “maintain their function, which occurs when they provide quality (safe, effective, patient-centered, timely, efficient, equitable) and continuous critical and essential services, amidst the crises, while leaving no one behind” (2, 21–24). Whereas historically public health functions have been associated with primary care; recent evidence on building resilient health systems to achieve UHC and health security highlights the contributions of all health systems actors (including hospitals) in fulfilling EPHF (13). This study confirmed that hospitals have a responsibility in fulfilling their essential public functions whether through health promotion and education, surveillance, risk reduction, or other activities which minimize the impacts of public health emergencies (25–27). Moreover, and consistently with the lessons from the global responses to Ebola and COVID-19, scholars concluded that the interplay between communities and hospitals particularly during emergencies is an essential part of the response and early recovery (28–30).

Secondly, across the EMR, hospital managers' expectations from national and global health institutions to enable their recovery from COVID-19 included: human resource management particularly regarding financial and logistical support, better leadership and coordination, and technical support through the provision of updated clinical evidence-based information. The qualitative findings of this study confirmed that hospitals cannot be resilient without the support of their community, health systems, and national and global public health institutions. Further confirming this interconnectedness, hospital resilience (and the role hospitals play in recovery) is vital to both community and health systems resilience (31, 32). Resilient hospitals integrated within a primary-care and whole-of-society approach, contribute and collaborate with different health and emergency response actors, including their community, MOH, and WHO, to fulfill their primary function of continuous delivery of essential services and secondary contributions in risk reduction, health promotion, and social and economic development (1, 2, 13, 31). Confirming the global literature, this study also found that the interconnections between hospitals and communities during health emergencies are essential to recovery as hospitals contribute majorly to the community's social, economic, and environmental development (33). Moreover, strengthening hospital resilience, particularly throughout the recovery phase, influences both policy and practice with implications across health, economic, social, and environmental domains. Furthermore, the lessons from the pandemic highlighted the need for more inclusive and community-oriented governance approaches (at both facility and national levels), including greater community engagement, gender-equal leadership, and fairer representation from marginalized communities to ensure that no one is left behind in BBB (34).

Thirdly, regarding evaluating hospital resilience before and during COVID-19: according to hospital managers and frontliners, despite a medium level of resilience to various types of hazards and generally high scores in response to non-COVID emergencies and disasters, the pandemic resulted in considerable changes in hospitals' resilience capacities. Hospital managers reflected that they learned to become better prepared to absorb various shocks but reflected lower levels of changes regarding their capacities to recover and transform. This is consistent with a systematic review of health systems resilience which found that the transformative capacity was the least researched or evaluated; indicating a significant gap in strategies to systematically evaluate the recovery stage (16). Despite these perceptions, scholars could argue that EMR hospitals' transformative and learning capacities increased as they adapted their systems and strategies in responding to and recovering from COVID-19. Further research is needed regarding institutionalizing learning across hospitals in the Region. Across the EMR, hospitals in resource-restrained and emergency-affected settings have exhibited an everyday resilience to a multitude of simultaneous hazards and chronic health systems shocks (e.g., societal, natural, and biological: civil unrest and instability, droughts or flooding, while managing COVID-19). In many of these settings, evaluating hospital resilience is nuanced by the different types of hazards; hospital managers reflected the challenges in the conceptualization of hospital resilience; as their hospitals may have been resilience to some types of hazards more than others, indicating a “partial” resilience which cannot be enumerated. Evaluating hospital resilience is complex given the multitude of qualitative and quantitative evaluation strategies and fragmented approaches presented in the empirical literature; this is especially difficult to do without a baseline assessment (2, 9, 35–37). Moreso, systematic reviews found that measuring or evaluating hospital (and health systems) resilience remains a fragmented and new topic in the empirical literature; qualitative approaches were found to be more comprehensive as quantitative ones were limited by the lack of objectivity and validated indices (2, 15, 16, 38–40).

Fourthly, regarding strengthening hospital resilience throughout the recovery phase; hospital managers highlighted the components of hospital resilience namely resilient staff, sustainable finance, and adaptive leadership and management. Firstly, qualitative findings from this study echoed global literature confirming that the ability to surge staff and redistribute health workers according to hospital needs was critical to the hospital's response, recovery, and ultimately resilience (6, 14, 24, 27, 28, 41, 42). Given the prolonged response and recovery phases of COVID-19 over the last 3 years, scaling up mental health services and psychosocial support as well as providing training on stress, time, and crisis- management is essential to recovery (2, 15, 43, 44). The COVID-19 pandemic has shown the importance of strengthening health workforce resilience as burnout threatens the retention, motivation, and mental health of frontliners and first responders as the world enters its third year of the pandemic (45, 46). Recent studies have shown that one in five health workers is leaving the health sector due to the increasing pressures and limited support (47). This is critical to consider in the EMR as most LMICs and countries in emergencies already suffer from severe shortages of health workers, including critical care and other emergency-related specialists (48). The psychological aspects of health workforce resilience and interventions related to self-care remain understudied especially in the EMR where their implications are most needed especially with the high number of humanitarian crises. Secondly, the lack of financial resources and flexible financing arrangements were raised as key challenges which inhibited hospitals from timely recovery, particularly in LMICs and FCS where centralized budgeting and donor-dependency are common (3, 8, 14). It is also crucial to differentiate financial resilience between private and public sector hospitals and their implications on the rapidity of their response and recovery. In many contexts, particularly following natural disasters, investments must be made to rebuild hospitals *stronger*, ensuring their hard resilience to enable their soft resilience (2, 17, 37, 39, 49). Further to this, one of the most critical elements of recovery was related to rapidity; building back *faster* with the needed financial and material resources to resume operations (10). Notably, the hospital's chief expectations of MOH and WHO were financial and material resources, especially in resource-restrained settings. These parallels between findings for study objectives 2 and 4 (the expectations of hospitals to enable recovery and the main lessons which allowed hospitals to be resilient) point to the need for resilient and decentralized financing mechanisms to enable recovery, consistent with global and regional literature (1, 7, 15, 27, 50). Operational guidance on strengthening hospital and health systems resilience detail specific interventions for securing and improving finance, logistics, and supply chain management throughout the recovery stages (1, 4). Thirdly, consistent with regional and global research, this study confirmed that strong leadership and coordination and strengthening learning mechanisms are required for recovery and resilience from emergencies, both at the facility and national levels (34, 51, 52). A study on hospital responses to COVID-19 from the Region found that the most frequently cited lessons included: “prevention,” “leadership,” “coordination,” “human resource management,” and “communication” (7). These lessons highlight the importance of strengthening hospitals' preparedness along with agile and adaptive leadership and management in health emergencies and DRM (36, 51, 53). Within the context of DRM, hospital managers and policymakers alike must proactively and innovatively plan, manage, and protect their human, financial, and material resources; these stakeholders would benefit from building learning organizations in recovering from COVID-19 and in preparation for future emergencies. Moreover, consistent with current evidence, strengthening the capacities of hospital managers in emergency response is critical to strengthening hospital resilience (51).

In the aftermath of COVID-19, the momentum for recovery and the impetus on BBB has highlighted the critical need to rebuild hospitals, health systems, and societies around the axes of **sustainability and equity**. On the one hand, environmental sustainability, rational use of resources, and minimizing wastage were minimally mentioned throughout the qualitative data, recent studies found that hospitals must play a significant role in mitigating their contributions to climate change. A recent WHO report found that medical waste from the COVID-19 response has strained already weak healthcare waste management systems as a third of healthcare facilities (two-thirds in the least developed countries) are not equipped to handle existing waste loads, not considering the additional waste load from the pandemic (54). As of the end of 2021, ~87,000 tons of personal protective equipment (PPE) were procured and shipped, 140 million test kits, generating 2,600 tons of mainly plastic waste and 731,000 L of chemical waste, and over 8 billion doses of vaccine have been administered globally producing 144,000 tons of additional waste in the form of syringes, needles, and safety boxes (55). Moreover, this type of pollution results in magnanimous environmental threats and health risks for health workers and vulnerabilities for communities living near landfills and disposal sites. The pandemic exposed poorly managed trade-offs between resuming services to mitigate financial losses, overuse of resources toward infection, prevention and control (IPC) measures, and few environmentally sustainable practices, highlighting the urgent need for a healthy and green recovery. Further to this, recognizing the impacts that hospitals and health facilities have on health and the environment, the WHO developed the *Guidance for Climate Resilient and Environmentally Sustainable Health Care Facilities* which ensures that health facilities are built to be environmentally sustainable by implementing interventions that optimize the consumption of resources (e.g., water, energy, food), reduce emissions of greenhouse gases, and properly manage waste (including biological, chemical and radiological) and are sustained through ethical and environmentally sustainable procurement of goods and services (56). On the other hand, the pandemic also exposed and exacerbated health, social, and economic inequalities, especially in conflict-affected settings as many in the EMR; in response, the Commission on Social Determinants of Health recommended a “Build Back Fairer” approach to ensure and enhance health equity in the post-pandemic recovery (57). The theme and sub-theme of equity were also minimally mentioned across the findings of this study beyond the use of telemedicine to reach vulnerable groups. This indicates the need for political, social, and multi-sectoral initiatives to ensure that no one is left behind in recovering from COVID-19.

Strengthening hospital resilience throughout the recovery phase not only improves efficiency and effectiveness in emergency response but also ensures continuity in the provision of critical and essential health services during emergencies and guarantees sustainable development in the health system. In the early phases of response and recovery, a report published by WHO in Aug 2020, found that low and lower-middle-income countries reported the highest percentage of partial disruptions in 75% of services essential health services during the COVID-19 pandemic where the EMR was the most affected Region (58). Notably, in the EMR, emergency and critical care were the least disrupted service group; a significant achievement, where more than half of countries face humanitarian emergencies, attributed to the resilience of hospitals, especially in the recovery phase. Strengthening emergency care systems during routine times is critical to a hospital's resilience during emergencies and to various types of hazards. Some studies even evaluate hospital resilience using the functionality and performance of hospital emergency departments during and prior to the onset of disasters; further highlighting the importance of hospitals' resilience in the response and early recovery stages (5, 6). Moreso, regional research found that in the EMR, hospitals consume around 70% of public health expenditures and employ the vast majority of health workers nationally (59). Interventions to strengthen health systems' resilience for public health emergencies, therefore, require a specific focus on strengthening and transforming hospital sectors. Ultimately, ensuring the recovery of hospitals and strengthening their resilience increases financial gains and economic growth at the individual, familial, community, and national levels. A study from the USA found that the national hospital sector supports 16 million total jobs and around $3 trillion in an economic activity where each hospital job supports 2 additional jobs and each dollar spent by a hospital contributes to $2.3 in additional businesses (60).

Finally, in operationalizing hospital and health systems resilience, it is imperative to consider the role of hospitals within PHC-oriented models of care (13, 59). Hospital resilience is intricately integrated within strengthening both health systems and community resilience; which are able to absorb, adapt, transform, and learn in the face of various types of hazards and shocks and respond to community needs both in routine times and emergencies (2). Recent evidence has pointed to the importance of context-appropriate coordination mechanisms to actualize a multisectoral whole of society approach to strengthening hospital and health systems resilience; this requires integrating various stakeholders such as UN, development partners and donors (especially in humanitarian settings), public health institutions, academia, private sector, hospitals and primary care (1, 7, 13, 59, 61). Moreover, building resilient health systems requires investing in EPHF to achieve UHC and health security (13). A recent regional analysis from the African Region highlighted the role of national public health institutions in EPHF for both UHC and DRM with limited mention of hospitals (61). Further research is needed to delineate the roles and functions of hospitals in implementing PHC-oriented models of care, fulfilling EPHF, and protecting health security through DRM.

One of the major strengths of this study is that it is among the first to capture hospitals' experiences responding, recovering, and building resilience during COVID-19 at a regional level. As the Region with the highest number of emergencies, the perspectives and lessons learned on recovery and resilience offer both context-specific insights along with practical approaches for hospitals in similar humanitarian and/or resource-restrained settings. This study addresses a gap in the regional and global evidence by exploring the roles that hospitals play in recovery and resilience, particularly from the perspective of frontliners and hospital managers. Additionally, this paper is among the first to capture the expectations hospital managers have of their ministries and WHO during public health emergencies, which provides invaluable lessons for national, regional, and global health and DRM policymakers and practitioners in anticipation of forthcoming public health emergencies. On the other hand, as this data was collected during the response to COVID-19, this study was limited by the high workload, pressures, and limited time of frontliners and hospital managers. The short study period also constrained the number, geographic distribution, and diversity of KIIs and survey respondents; whose individual experiences do not reflect all hospitals (size, public, private, peripheral, or central) of a country. The self-reporting bias presents a limitation to the survey tool whereby it is likely that respondents report a higher score than anticipated, reflecting a more positive evaluation of their hospital responses, recovery, and resilience. Nevertheless, the triangulation with other survey questions including open-ended ones, as well as with key informant interviews provided a more complete picture regarding hospital resilience capacities, lessons, and challenges in the EMR. Furthermore, the topic of hospital resilience, and the hospital's role in recovery, health systems for health security, and sustainable development, remain nascent and require further research, particularly from the Global South, humanitarian, and resource-restrained settings. Systematic reviews on both hospital and health system resilience highlight the limited evidence on this new subject along with the diversity and discrepancies between its conceptualization, operationalization, and evaluation (2, 16, 38). The exact impacts that hospitals play in ensuring health systems fulfill their essential public health functions remains understudied and requires further investigation (62). Additional research is also needed regarding scaling adaptive and agile hospital management along with the costs, specific interventions, and evaluations of hospital resilience (including hospital workforce, supply chain/logistics/financial resilience, etc…).

## 5. Conclusion

During emergencies, hospitals are among the community's first points of contact with health systems, it is, therefore, critical to ensure their functionality across the response and recovery stages of DRM. COVID-19 showed that hospitals played a critical role in service delivery and contributed to EPHF, health systems resilience, health security, and sustainable social, economic, and environmental development. Policymakers and hospital managers should be equipped with operational guides and tools to continuously improve hospital resilience in preparation for future outbreaks and other public health emergencies. Strengthening hospital resilience requires investing in hospital workers and their wellbeing, innovative and flexible mechanisms for resource mobilization, especially in resource-restrained settings, and finally, agile, adaptive, and proactive leadership and coordination.

## Data availability statement

The original contributions presented in the study are included in the article/Supplementary material, further inquiries can be directed to the corresponding author.

## Ethics statement

The studies involving human participants were reviewed and approved by WHO EMRO Regional Ethics Committee. The patients/participants provided their written informed consent to participate in this study.

## Author contributions

MK and HR: conceptualization, data collection, data analysis, and writing and editing. JA-B, AN, AA, and HK: revision of data collection tools, data collection, data analysis, and technical revision of manuscript. All authors read and revised the final version.
